# Bis(dicyanamido-κ*N*)[tris­(3-amino­propyl)amine-κ^4^ *N*]nickel(II)

**DOI:** 10.1107/S1600536813015651

**Published:** 2013-06-15

**Authors:** Jun Luo, Xin-Rong Zhang, Li-Juan Qiu, Feng Yang, Bao-Shu Liu

**Affiliations:** a School of Pharmacy, Second Military Medical University, Shanghai 200433, People’s Republic of China

## Abstract

In the title complex, [Ni(C_2_N_3_)_2_(C_9_H_24_N_4_)], the Ni^II^ atom is coordinated in a distorted octa­hedral geometry by one tris­(3-amino­prop­yl)amine (tris­apa) ligand and two dicyanamide (dca) ligands [one of them disordered in a 0.681 (19):0319 (19) ratio]. Inter­molecular N—H⋯N hydrogen bonds involving the N atoms of the dca anions and the tris­apa amine H atoms result in the formation of a three-dimensional network.

## Related literature

For magnetic properties and structural types of dicyanamide complexes, see: Batten (2005 ▶); Batten & Murray (2003 ▶); Batten *et al.* (1998 ▶); Ghosh *et al.* (2011 ▶); Ion *et al.* (2013 ▶); Manson *et al.* (1999 ▶); Mastropietro *et al.* (2013 ▶); Turner *et al.* (2011 ▶). For dicyanamide complexes with multidentate Schiff bases, see: Sadhukhan *et al.* (2011 ▶); Fondo *et al.* (2011 ▶); Bhar *et al.* (2011 ▶). For dicyanamide complexes with polyamines as co-ligands, see: Khan *et al.* (2011 ▶). For Ni—N bond lengths in aliphatic amine nickel complexes, see: Cho *et al.* (2002 ▶); Brezina *et al.* (1999 ▶) and in [Ni(tn)_2_{C_2_N_3_}](ClO_4_)(tn is tri­methyl­enedi­amine, see: Li *et al.* (2002 ▶). 
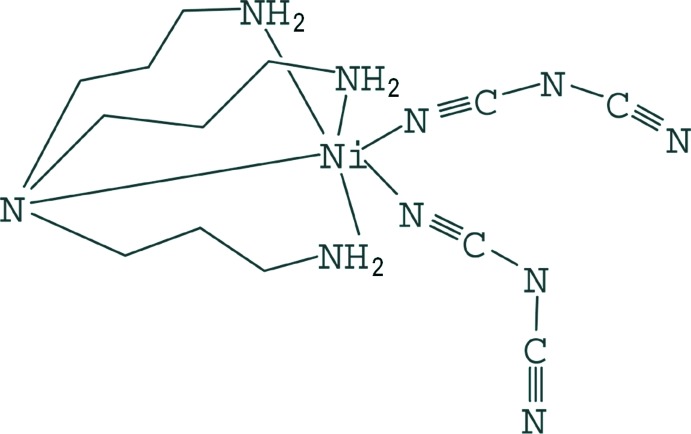


## Experimental

### 

#### Crystal data

[Ni(C_2_N_3_)_2_(C_9_H_24_N_4_)]
*M* *_r_* = 379.13
Monoclinic, 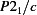

*a* = 10.171 (1) Å
*b* = 11.3960 (11) Å
*c* = 15.5305 (15) Å
β = 105.660 (2)°
*V* = 1733.3 (3) Å^3^
*Z* = 4
Mo *K*α radiation
μ = 1.14 mm^−1^
*T* = 213 K
0.17 × 0.09 × 0.05 mm

#### Data collection

Bruker SMART APEX CCD area-detector diffractometer
Absorption correction: multi-scan (*SADABS*; Bruker, 2000 ▶) *T*_min_ = 0.830, *T*_max_ = 0.945
12722 measured reflections
4056 independent reflections
3403 reflections with *I* > 2σ(*I*)
*R*_int_ = 0.024

#### Refinement

*R*[*F* ^2^ > 2σ(*F* ^2^)] = 0.027
*wR*(*F* ^2^) = 0.079
*S* = 1.07
4056 reflections
269 parameters
20 restraints
H atoms treated by a mixture of independent and constrained refinement
Δρ_max_ = 0.38 e Å^−3^
Δρ_min_ = −0.32 e Å^−3^

### 

Data collection: *SMART* (Bruker, 2000 ▶); cell refinement: *SAINT* (Bruker, 2000 ▶); data reduction: *SAINT*; program(s) used to solve structure: *SHELXS97* (Sheldrick, 2008 ▶); program(s) used to refine structure: *SHELXL97* (Sheldrick, 2008 ▶); molecular graphics: *SHELXTL* (Sheldrick, 2008 ▶); software used to prepare material for publication: *SHELXTL*.

## Supplementary Material

Crystal structure: contains datablock(s) global, I. DOI: 10.1107/S1600536813015651/bg2508sup1.cif

Structure factors: contains datablock(s) I. DOI: 10.1107/S1600536813015651/bg2508Isup2.hkl

Additional supplementary materials:  crystallographic information; 3D view; checkCIF report

## Figures and Tables

**Table 1:** Hydrogen-bond geometry (Å, °)

*D*—H⋯*A*	*D*—H	H⋯*A*	*D*⋯*A*	*D*—H⋯*A*
N2—H2*C*⋯N10^i^	0.92 (2)	2.38 (2)	3.255 (2)	160 (2)
N2—H2*D*⋯N10^ii^	0.80 (2)	2.43 (2)	3.193 (2)	158 (2)
N3—H3*D*⋯N10^i^	0.90 (2)	2.36 (2)	3.154 (2)	148 (2)
N4—H4*D*⋯N7^iii^	0.90 (3)	2.19 (3)	3.094 (3)	176 (2)

Symmetry codes: (i) 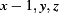; (ii) 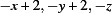; (iii) 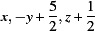.

**Fig. 1.**
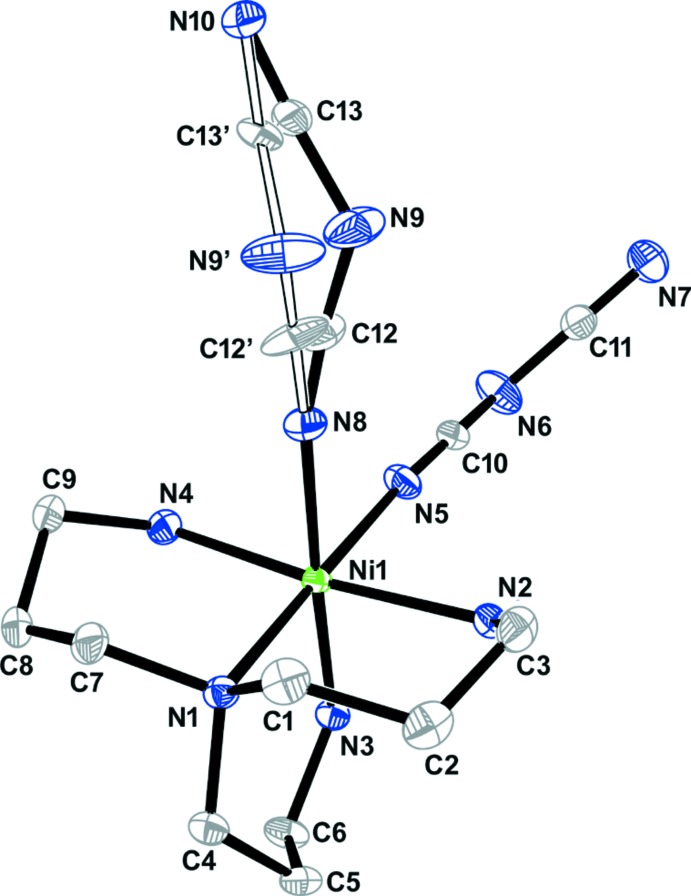
View of the molecule of I showing the atom-labelling scheme. Displacement ellipsoids are drawn at the 50% probability level. In open bonds, the minor disordered part of the molecule. H atoms not shown, for clarity.

**Fig. 2.**
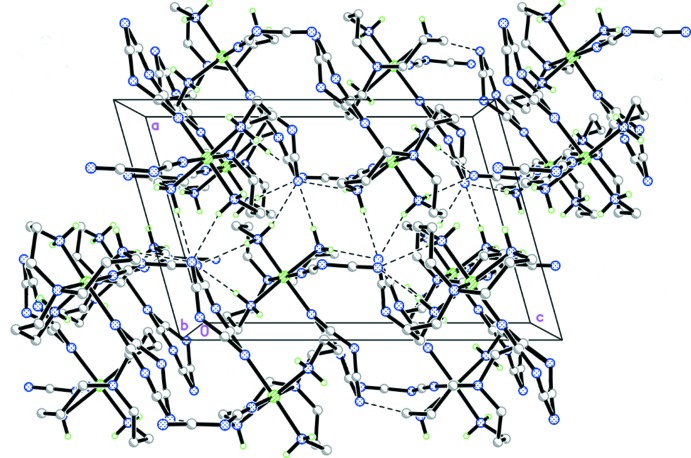
Three dimensional network in I formed by hydrogen-bonding interactions.

## supplementary crystallographic information

### Comment

Recently, dicyanamide complexes have attracted considerable interest because of their fascinating magnetic properties and diverse structural types (Turner *et al.*, 2011; Batten *et al.*, 2005; Batten *et al.*, 2003). For example, the binary transition metal dicyanamide complexes display long-range magnetic ordering, with the nature of the ordering dependent on the particular metal ion involved. Thus the Cr (47 K) and Mn (16 K) compounds are antiferromagnets (Manson *et al.*, 1999), while the Co (9 K) and Ni systems (21 K) are ferromagents (Batten *et al.*, 1998). It is well known that the structure and the magnetic property of the complexes are related to the nature of the co-ligands (Ghosh *et al.*, 2011; Mastropietro *et al.*, 2013; Ion *et al.*, 2013). Although a great effort is focused on studies of dicyanamide complexes with multidentate schiff bases (Sadhukhan *et al.*, 2011; Fondo *et al.*, 2011; Bhar *et al.*, 2011), few dicyanamide complexes with polyamines as co-ligands have been reported recently (Khan *et al.*, 2011). To further study the effect of the nature of co-ligands on the structures and properties of dicyanamide complexes, we herein report the synthesis and crystal structure of the title new nickel dicyanamide complex [Ni(trisapa)(C_2_N_3_)_2_] (I).

The nickel ion in I is coordinated by four N atoms from the tris(3-aminopropyl) amine and two terminal N atoms from two dicyanamide anions to form a distorted octahedral geometry, in which the equatorial plane is formed by the three N atoms(N2, N3, N4) of tris(3-aminopropyl)amine and one nitrile N atom (N8) of a monodentate (disordered) dicyanamide, where the disorder atoms are C12 and C12', N9 and N9', C13 and C13' respectively. The two apical sites are occupied by one trisapa N atom(N1) and one nitrile N atom (N5) of another monodentate dicyanamide (Fig. 1). Table. 2 shows the intermolecular hydrogen interactions between the uncoordinated N atoms of dicyanamide anions and the amine H atoms of trisapa, responsible of the construction of a three-dimensional network (Fig. 2). The Ni—*N* (trisapa) distances (2.100 (2)–2.196 (1) Å) are rather different, with values similar to the corresponding distances in the aliphatic amine nickel complexes (Cho *et al.*, 2002; Brezina *et al.*, 1999). The apical Ni—*N* (dicyanamide) distance(2.145 (1) Å) is slightly longer than the basal Ni—*N*(dicyanamide) distance(2.090 (2) Å). These distances in I are comparable to the corresponding ones in [Ni(tn)_2_{C_2_N_3_}](ClO_4_)(tn is trimethylenediamine, Li *et al.*, 2002). In I, N—Ni—N cis angles range from 89.36 (7)° to 90.37 (6)° (basal-basal) and 84.32 (6)° to 95.61 (6)° (basal-apical), indicating that the distortion from an ideal octahedral geometry in I is not serious.

### Experimental

A 4 ml ethanol solution of tris(3-aminopropyl)amine(0.10 mmol, 18.83 mg) and a 4 ml e thanol solution of nickel nitrate(0.10 mmol, 29.08 mg) were mixed and stirred for 5 min, the mixed solution was pale-blue. To the mixture was added a 2 ml aqueous solution of sodium dicyanamide (0.20 mmol, 17.81 mg). After stirred for another 5 min, the solution was filtered and the filtrate was slowly evaporated in air. After one week, blue block crystals of I were isolated in 34% yield. Anal: Calculated for C13H24N10Ni: C 41.18%, H 6.38%, N 36.95%. Found C 40.86%, H 6.47%, N 37.07%.

### Refinement

One of the dicyanamide units is disordered in two halves, which were refined with restraints (both metric as in displacement factors). The corresponding occupation factors refined to 0.681/0.319 (19). The amine H atom were found from difference maps and refined freely with a final N—H range 0.80 (2) Å - 0.92 (2) Å. Remaining H atoms were placed in geometrically idealized positions and constrained to ride on their parent atoms with C—H distances of 0.98 Å and U_iso_(H) = 1.2 × U(Host) .

### Crystal data

**Table:**

[Ni(C_2_N_3_)_2_(C_9_H_24_N_4_)]	*F*(000) = 800
*M**_r_* = 379.13	*D*_x_ = 1.453 Mg m^−^^3^
Monoclinic, *P*2_1_/*c*	Mo *K*α radiation, λ = 0.71073 Å
Hall symbol: -P 2ybc	Cell parameters from 5066 reflections
*a* = 10.171 (1) Å	θ = 2.3–27.7°
*b* = 11.3960 (11) Å	µ = 1.14 mm^−^^1^
*c* = 15.5305 (15) Å	*T* = 213 K
β = 105.660 (2)°	Block, blue
*V* = 1733.3 (3) Å^3^	0.17 × 0.09 × 0.05 mm
*Z* = 4	

### Data collection

**Table:**

Bruker SMART APEX CCD area-detector diffractometer	4056 independent reflections
Radiation source: fine-focus sealed tube	3403 reflections with *I* > 2σ(*I*)
Graphite monochromator	*R*_int_ = 0.024
φ and ω scans	θ_max_ = 27.8°, θ_min_ = 2.1°
Absorption correction: multi-scan (*SADABS*; Bruker, 2000)	*h* = −11→13
*T*_min_ = 0.830, *T*_max_ = 0.945	*k* = −14→14
12722 measured reflections	*l* = −20→19

### Refinement

**Table:**

Refinement on *F*^2^	Primary atom site location: structure-invariant direct methods
Least-squares matrix: full	Secondary atom site location: difference Fourier map
*R*[*F*^2^ > 2σ(*F*^2^)] = 0.027	Hydrogen site location: inferred from neighbouring sites
*wR*(*F*^2^) = 0.079	H atoms treated by a mixture of independent and constrained refinement
*S* = 1.07	*w* = 1/[σ^2^(*F*_o_^2^) + (0.0475*P*)^2^ + 0.0784*P*] where *P* = (*F*_o_^2^ + 2*F*_c_^2^)/3
4056 reflections	(Δ/σ)_max_ = 0.006
269 parameters	Δρ_max_ = 0.38 e Å^−^^3^
20 restraints	Δρ_min_ = −0.32 e Å^−^^3^

### Special details

**Table:**

Geometry. All e.s.d.'s (except the e.s.d. in the dihedral angle between two l.s. planes) are estimated using the full covariance matrix. The cell e.s.d.'s are taken into account individually in the estimation of e.s.d.'s in distances, angles and torsion angles; correlations between e.s.d.'s in cell parameters are only used when they are defined by crystal symmetry. An approximate (isotropic) treatment of cell e.s.d.'s is used for estimating e.s.d.'s involving l.s. planes.
Refinement. Refinement of *F*^2^ against ALL reflections. The weighted *R*-factor *wR* and goodness of fit *S* are based on *F*^2^, conventional *R*-factors *R* are based on *F*, with *F* set to zero for negative *F*^2^. The threshold expression of *F*^2^ > σ(*F*^2^) is used only for calculating *R*-factors(gt) *etc*. and is not relevant to the choice of reflections for refinement. *R*-factors based on *F*^2^ are statistically about twice as large as those based on *F*, and *R*- factors based on ALL data will be even larger.

### Fractional atomic coordinates and isotropic or equivalent isotropic displacement parameters (Å^2^)

**Table:**

	*x*	*y*	*z*	*U*_iso_*/*U*_eq_	Occ. (<1)
Ni1	0.75943 (2)	0.944842 (17)	0.199688 (12)	0.01927 (8)	
N1	0.78081 (14)	0.76691 (11)	0.25612 (9)	0.0232 (3)	
N2	0.63129 (16)	0.88585 (14)	0.07694 (9)	0.0274 (3)	
H2C	0.541 (2)	0.8859 (18)	0.0774 (13)	0.034 (5)*	
H2D	0.636 (2)	0.9404 (16)	0.0450 (14)	0.024 (5)*	
N3	0.58711 (15)	0.98960 (13)	0.24394 (10)	0.0230 (3)	
H3C	0.605 (2)	1.0556 (15)	0.2735 (13)	0.020 (5)*	
H3D	0.525 (2)	1.0082 (19)	0.1927 (15)	0.035 (5)*	
N4	0.88525 (17)	1.02601 (14)	0.31382 (11)	0.0296 (3)	
H4C	0.921 (2)	1.0799 (19)	0.2941 (15)	0.038 (6)*	
H4D	0.829 (2)	1.0540 (17)	0.3450 (16)	0.038 (6)*	
N5	0.73577 (17)	1.11767 (13)	0.14290 (10)	0.0325 (3)	
N6	0.6910 (2)	1.31289 (14)	0.07638 (12)	0.0460 (4)	
N7	0.7027 (2)	1.38147 (16)	−0.07076 (12)	0.0514 (5)	
N8	0.93047 (16)	0.91772 (14)	0.15190 (11)	0.0336 (3)	
N10	1.32388 (17)	0.95680 (15)	0.08290 (11)	0.0370 (4)	
C1	0.7900 (2)	0.67980 (16)	0.18584 (13)	0.0344 (4)	
H1A	0.8050	0.6020	0.2137	0.041*	
H1B	0.8709	0.6988	0.1656	0.041*	
C2	0.6680 (2)	0.67200 (16)	0.10390 (13)	0.0373 (4)	
H2A	0.6733	0.5982	0.0726	0.045*	
H2B	0.5842	0.6700	0.1235	0.045*	
C3	0.6591 (2)	0.77285 (18)	0.03917 (12)	0.0372 (4)	
H3A	0.7453	0.7786	0.0227	0.045*	
H3B	0.5864	0.7567	−0.0154	0.045*	
C4	0.67169 (19)	0.72696 (15)	0.29801 (12)	0.0304 (4)	
H4A	0.7062	0.7383	0.3628	0.037*	
H4B	0.6595	0.6423	0.2876	0.037*	
C5	0.53150 (19)	0.78395 (14)	0.26810 (12)	0.0285 (4)	
H5A	0.5000	0.7812	0.2027	0.034*	
H5B	0.4674	0.7378	0.2914	0.034*	
C6	0.5276 (2)	0.90978 (15)	0.29799 (13)	0.0318 (4)	
H6A	0.4328	0.9326	0.2925	0.038*	
H6B	0.5786	0.9166	0.3611	0.038*	
C7	0.91496 (19)	0.75441 (16)	0.32628 (13)	0.0336 (4)	
H7A	0.9885	0.7577	0.2965	0.040*	
H7B	0.9179	0.6764	0.3533	0.040*	
C8	0.9448 (2)	0.84521 (17)	0.40100 (12)	0.0374 (4)	
H8A	0.8609	0.8602	0.4188	0.045*	
H8B	1.0125	0.8130	0.4530	0.045*	
C9	0.9974 (2)	0.96002 (16)	0.37472 (13)	0.0350 (4)	
H9A	1.0369	1.0069	0.4284	0.042*	
H9B	1.0693	0.9446	0.3450	0.042*	
C10	0.71677 (18)	1.20685 (15)	0.10710 (11)	0.0271 (4)	
C11	0.6993 (2)	1.34348 (16)	−0.00333 (13)	0.0342 (4)	
N9	1.0877 (5)	0.8948 (10)	0.0568 (4)	0.066 (2)	0.681 (19)
C12	1.0075 (9)	0.9122 (9)	0.1113 (6)	0.0352 (18)	0.681 (19)
C13	1.2146 (8)	0.9299 (11)	0.0737 (6)	0.0328 (15)	0.681 (19)
N9'	1.1203 (14)	0.8524 (7)	0.1020 (17)	0.063 (4)	0.319 (19)
C12'	1.027 (2)	0.8940 (19)	0.1275 (14)	0.048 (6)	0.319 (19)
C13'	1.2234 (14)	0.915 (2)	0.0963 (13)	0.031 (3)	0.319 (19)

### Atomic displacement parameters (Å^2^)

**Table:**

	*U*^11^	*U*^22^	*U*^33^	*U*^12^	*U*^13^	*U*^23^
Ni1	0.01766 (12)	0.02156 (12)	0.01955 (12)	−0.00006 (8)	0.00666 (8)	0.00235 (7)
N1	0.0205 (7)	0.0213 (6)	0.0279 (7)	0.0009 (5)	0.0069 (5)	0.0020 (5)
N2	0.0251 (8)	0.0366 (8)	0.0214 (7)	−0.0007 (6)	0.0077 (6)	0.0019 (6)
N3	0.0240 (7)	0.0213 (7)	0.0256 (7)	0.0011 (6)	0.0100 (6)	0.0000 (6)
N4	0.0286 (8)	0.0293 (8)	0.0288 (8)	−0.0061 (7)	0.0039 (6)	0.0000 (6)
N5	0.0375 (9)	0.0319 (8)	0.0303 (8)	0.0003 (7)	0.0131 (7)	0.0089 (6)
N6	0.0673 (13)	0.0314 (8)	0.0454 (10)	0.0115 (8)	0.0256 (9)	0.0137 (7)
N7	0.0540 (12)	0.0509 (11)	0.0462 (10)	−0.0011 (9)	0.0080 (9)	0.0253 (9)
N8	0.0250 (8)	0.0422 (8)	0.0369 (8)	−0.0008 (7)	0.0139 (7)	0.0003 (7)
N10	0.0255 (9)	0.0523 (10)	0.0350 (9)	−0.0021 (7)	0.0113 (7)	−0.0009 (7)
C1	0.0341 (10)	0.0251 (9)	0.0456 (11)	0.0076 (7)	0.0135 (8)	−0.0032 (7)
C2	0.0378 (11)	0.0320 (9)	0.0435 (10)	−0.0030 (8)	0.0134 (8)	−0.0172 (8)
C3	0.0337 (10)	0.0519 (11)	0.0271 (9)	−0.0036 (9)	0.0103 (8)	−0.0150 (8)
C4	0.0333 (10)	0.0267 (8)	0.0323 (9)	−0.0058 (7)	0.0106 (7)	0.0050 (7)
C5	0.0291 (9)	0.0268 (8)	0.0337 (9)	−0.0078 (7)	0.0156 (7)	−0.0030 (7)
C6	0.0398 (11)	0.0269 (8)	0.0366 (10)	−0.0033 (8)	0.0241 (8)	−0.0021 (7)
C7	0.0253 (9)	0.0320 (9)	0.0397 (10)	0.0043 (7)	0.0022 (8)	0.0099 (8)
C8	0.0323 (10)	0.0454 (11)	0.0281 (9)	−0.0045 (8)	−0.0029 (7)	0.0113 (8)
C9	0.0279 (10)	0.0427 (11)	0.0292 (9)	−0.0070 (8)	−0.0013 (7)	0.0043 (7)
C10	0.0255 (9)	0.0330 (9)	0.0248 (8)	−0.0011 (7)	0.0104 (7)	0.0034 (7)
C11	0.0291 (10)	0.0302 (9)	0.0405 (10)	0.0000 (7)	0.0046 (8)	0.0097 (8)
N9	0.039 (2)	0.117 (5)	0.051 (3)	−0.030 (2)	0.029 (2)	−0.044 (3)
C12	0.023 (3)	0.045 (5)	0.040 (2)	−0.003 (2)	0.013 (2)	−0.012 (2)
C13	0.031 (2)	0.050 (4)	0.021 (3)	−0.0063 (18)	0.0128 (17)	−0.008 (3)
N9'	0.055 (5)	0.040 (4)	0.117 (10)	−0.004 (3)	0.062 (6)	−0.018 (4)
C12'	0.021 (6)	0.018 (4)	0.107 (14)	−0.006 (4)	0.021 (8)	−0.005 (6)
C13'	0.034 (5)	0.044 (6)	0.025 (8)	0.003 (4)	0.025 (5)	−0.005 (6)

### Geometric parameters (Å, º)

**Table:**

Ni1—N8	2.090 (2)	C1—H1A	0.9800
Ni1—N4	2.100 (2)	C1—H1B	0.9800
Ni1—N2	2.108 (1)	C2—C3	1.513 (3)
Ni1—N3	2.111 (1)	C2—H2A	0.9800
Ni1—N5	2.145 (1)	C2—H2B	0.9800
Ni1—N1	2.196 (1)	C3—H3A	0.9800
N1—C1	1.497 (2)	C3—H3B	0.9800
N1—C4	1.501 (2)	C4—C5	1.521 (3)
N1—C7	1.506 (2)	C4—H4A	0.9800
N2—C3	1.474 (2)	C4—H4B	0.9800
N2—H2C	0.92 (2)	C5—C6	1.511 (2)
N2—H2D	0.80 (2)	C5—H5A	0.9800
N3—C6	1.474 (2)	C5—H5B	0.9800
N3—H3C	0.87 (2)	C6—H6A	0.9800
N3—H3D	0.90 (2)	C6—H6B	0.9800
N4—C9	1.476 (2)	C7—C8	1.523 (3)
N4—H4C	0.82 (2)	C7—H7A	0.9800
N4—H4D	0.90 (3)	C7—H7B	0.9800
N5—C10	1.150 (2)	C8—C9	1.511 (3)
N6—C10	1.300 (2)	C8—H8A	0.9800
N6—C11	1.311 (3)	C8—H8B	0.9800
N7—C11	1.142 (3)	C9—H9A	0.9800
N8—C12	1.132 (6)	C9—H9B	0.9800
N8—C12'	1.18 (1)	N9—C13	1.309 (8)
N10—C13	1.124 (7)	N9—C12	1.339 (7)
N10—C13'	1.20 (1)	N9'—C12'	1.22 (2)
C1—C2	1.522 (3)	N9'—C13'	1.29 (2)
			
N8—Ni1—N4	89.36 (7)	C1—C2—H2B	108.8
N8—Ni1—N2	90.14 (6)	H2A—C2—H2B	107.7
N4—Ni1—N2	172.26 (6)	N2—C3—C2	112.5 (1)
N8—Ni1—N3	174.36 (6)	N2—C3—H3A	109.1
N4—Ni1—N3	89.38 (7)	C2—C3—H3A	109.1
N2—Ni1—N3	90.37 (6)	N2—C3—H3B	109.1
N8—Ni1—N5	90.09 (6)	C2—C3—H3B	109.1
N4—Ni1—N5	85.28 (6)	H3A—C3—H3B	107.8
N2—Ni1—N5	87.00 (6)	N1—C4—C5	118.7 (1)
N3—Ni1—N5	84.32 (6)	N1—C4—H4A	107.6
N8—Ni1—N1	90.16 (6)	C5—C4—H4A	107.6
N4—Ni1—N1	95.61 (6)	N1—C4—H4B	107.6
N2—Ni1—N1	92.11 (6)	C5—C4—H4B	107.6
N3—Ni1—N1	95.43 (5)	H4A—C4—H4B	107.1
N5—Ni1—N1	179.08 (6)	C6—C5—C4	114.3 (2)
C1—N1—C4	108.3 (1)	C6—C5—H5A	108.7
C1—N1—C7	104.0 (1)	C4—C5—H5A	108.7
C4—N1—C7	106.8 (1)	C6—C5—H5B	108.7
C1—N1—Ni1	109.9 (1)	C4—C5—H5B	108.7
C4—N1—Ni1	116.6 (1)	H5A—C5—H5B	107.6
C7—N1—Ni1	110.4 (1)	N3—C6—C5	111.2 (1)
C3—N2—Ni1	120.0 (1)	N3—C6—H6A	109.4
C3—N2—H2C	108 (1)	C5—C6—H6A	109.4
Ni1—N2—H2C	112 (1)	N3—C6—H6B	109.4
C3—N2—H2D	112 (1)	C5—C6—H6B	109.4
Ni1—N2—H2D	101 (1)	H6A—C6—H6B	108.0
H2C—N2—H2D	103 (2)	N1—C7—C8	116.3 (2)
C6—N3—Ni1	122.8 (1)	N1—C7—H7A	108.2
C6—N3—H3C	107 (1)	C8—C7—H7A	108.2
Ni1—N3—H3C	108 (1)	N1—C7—H7B	108.2
C6—N3—H3D	111 (1)	C8—C7—H7B	108.2
Ni1—N3—H3D	102 (1)	H7A—C7—H7B	107.4
H3C—N3—H3D	105 (2)	C9—C8—C7	113.3 (2)
C9—N4—Ni1	120.4 (1)	C9—C8—H8A	108.9
C9—N4—H4C	106 (2)	C7—C8—H8A	108.9
Ni1—N4—H4C	104 (2)	C9—C8—H8B	108.9
C9—N4—H4D	109 (1)	C7—C8—H8B	108.9
Ni1—N4—H4D	106 (1)	H8A—C8—H8B	107.7
H4C—N4—H4D	111 (2)	N4—C9—C8	110.2 (2)
C10—N5—Ni1	175.1 (2)	N4—C9—H9A	109.6
C10—N6—C11	122.4 (2)	C8—C9—H9A	109.6
C12—N8—Ni1	166.7 (6)	N4—C9—H9B	109.6
C12'—N8—Ni1	175 (1)	C8—C9—H9B	109.6
N1—C1—C2	116.8 (2)	H9A—C9—H9B	108.1
N1—C1—H1A	108.1	N5—C10—N6	172.1 (2)
C2—C1—H1A	108.1	N7—C11—N6	172.9 (2)
N1—C1—H1B	108.1	C13—N9—C12	124.3 (7)
C2—C1—H1B	108.1	N8—C12—N9	172.3 (9)
H1A—C1—H1B	107.3	N10—C13—N9	175 (1)
C3—C2—C1	113.6 (2)	C12'—N9'—C13'	122 (2)
C3—C2—H2A	108.8	N8—C12'—N9'	170 (2)
C1—C2—H2A	108.8	N10—C13'—N9'	169 (2)
C3—C2—H2B	108.8		

### Hydrogen-bond geometry (Å, º)

**Table:**

*D*—H···*A*	*D*—H	H···*A*	*D*···*A*	*D*—H···*A*
N2—H2*C*···N10^i^	0.92 (2)	2.38 (2)	3.255 (2)	160 (2)
N2—H2*D*···N10^ii^	0.80 (2)	2.43 (2)	3.193 (2)	158 (2)
N3—H3*D*···N10^i^	0.90 (2)	2.36 (2)	3.154 (2)	148 (2)
N4—H4*D*···N7^iii^	0.90 (3)	2.19 (3)	3.094 (3)	176 (2)

Symmetry codes: (i) *x*−1, *y*, *z*; (ii) −*x*+2, −*y*+2, −*z*; (iii) *x*, −*y*+5/2, *z*+1/2.
